# Taxonomic and Functional Distribution of Bacterial Communities in Domestic and Hospital Wastewater System: Implications for Public and Environmental Health

**DOI:** 10.3390/antibiotics10091059

**Published:** 2021-09-01

**Authors:** Ramganesh Selvarajan, Timothy Sibanda, Jeevan Pandian, Kevin Mearns

**Affiliations:** 1 Department of Environmental Sciences, College of Agricultural and Environmental Sciences, UNISA, Florida 1709, South Africa; mearnkf@unisa.ac.za; 2 Department of Biochemistry, Microbiology and Biotechnology, University of Namibia, Mandume Ndemufayo Ave, Pionierspark, Windhoek 13301, Namibia; timsibanda@gmail.com; 3 P.G and Research Department of Microbiology, J.J College of Arts and Science (Autonomous), Pudukkottai 622422, Tamil Nadu, India; pjjeevanmicro@gmail.com

**Keywords:** antimicrobials, antibiotic resistance genes, untreated wastewater, public health, environmental health

## Abstract

The discharge of untreated hospital and domestic wastewater into receiving water bodies is still a prevalent practice in developing countries. Unfortunately, because of an ever-increasing population of people who are perennially under medication, these wastewaters contain residues of antibiotics and other antimicrobials as well as microbial shedding, the direct and indirect effects of which include the dissemination of antibiotic resistance genes and an increase in the evolution of antibiotic-resistant bacteria that pose a threat to public and environmental health. This study assessed the taxonomic and functional profiles of bacterial communities, as well as the antibiotic concentrations in untreated domestic wastewater (DWW) and hospital wastewater (HWW), using high-throughput sequencing analysis and solid-phase extraction coupled to Ultra-high-performance liquid chromatography Mass Spectrometry (UHPLC–MS/MS) analysis, respectively. The physicochemical qualities of both wastewater systems were also determined. The mean concentration of antibiotics and the concentrations of Cl^−^, F^−^ and PO_4_^3^ were higher in HWW samples than in DWW samples. The phylum *Firmicutes* was dominant in DWW with a sequence coverage of 59.61% while *Proteobacteria* was dominant in HWW samples with a sequence coverage of 86.32%. At genus level, the genus *Exiguobacterium* (20.65%) and *Roseomonas* (67.41%) were predominant in DWW and HWW samples, respectively. Several pathogenic or opportunistic bacterial genera were detected in HWW (*Enterococcus, Pseudomonas* and *Vibrio*) and DWW (*Clostridium*, *Klebsiella, Corynebacterium, Bordetella, Staphylocccus* and *Rhodococcus*) samples. Functional prediction analysis indicated the presence of beta-lactam resistance, cationic antimicrobial peptide (CAMP) resistance and vancomycin resistance genes in HWW samples. The presence of these antibiotic resistance genes and cassettes were positively correlated with the presence of pathogens. These findings show the risk posed to public and environmental health by the discharge of untreated domestic and hospital wastewaters into environmental water bodies.

## 1. Introduction

Wastewater originates from various anthropogenic sources including mining and agricultural activities, as well as domestic, industrial, and hospital effluents [1,2]. Typically, domestic wastewater (DWW) is characterised by high amounts of organic load that provides suitable substrate to the growth and/or survival of a wide range of microorganisms including bacteria, viruses and protozoa [3]. Compared to DWW effluents, hospital wastewater (HWW) effluents contain, in addition to organic load, high concentrations of disinfectants, microbial shedding as well as partially metabolised antibiotics and other pharmaceuticals originating from patient excrement [1,4,5]. In cases where wastewater treatment technologies are inadequate to completely remove these pharmaceuticals and microbial shedding from wastewater before discharge, they pose a threat as environmental reservoirs of water and soil-borne pathogens and antibiotic resistance genes (ARGs).

There still remain loopholes in the regulation and use of antibiotics in many developing countries, including India, where these drugs are still available over the counter without a doctor’s prescription [4,5,6]. This escalates the evolution and spread of resistant pathogenic bacterial strains in the given ecosystem [7,8]. Consequently, wastewater effluents and, in particular, HWW effluents, have come to be considered an important driver for the selection of antibiotic resistance genes (ARGs) in between pathogenic and non-pathogenic microbes [9]. This exchange and/or transmission of ARGs in the environment occurs predominantly through mutations and horizontal gene transfer (HGT) mechanism via mobile genetic elements (MGEs) such as integrons that are associated to insertion elements, transposons and plasmids [10,11,12]. In addition, physical forces such as wind and water movement can also move pathogenic and/or drug resistance bacteria over large distances and facilitate the dissemination of resistant bacteria in the wide environment [13]. The selection for antibiotic resistance has broad epidemiological consequences [14], particularly in terms of an accelerated rate of evolution of multiple drug/antibiotic resistant bacteria, as it runs in the face of a decelerated rate of discovery of novel antibiotics. This poses a significant public and environmental health ticking time-bomb that humanity cannot any longer afford to sit and watch unravel.

Known bacterial pathogens found in untreated wastewater from hospitals and other health care units include those from the phyla *Proteobacteria, Firmicutes, Bacteroidetes, Chloroflexi, Planctomycetes, Acidobacteria* and *Spirochetes* [15,16,17], and mostly of the genus *Acinetobacter*, *Aeromonas*, *Shigella*, *Klebsiella*, *Stenotrophomonas*, *Enterobacter*, *Escherichia*, *Pseudomonas*, *Vibrio*, *Staphylococcus*, *Clostridium* and *Ralstonia* [18,19,20,21]. A survey based on different untreated HWW effluents put the detection rate of pathogenic bacterial species at between 14.6% and 33.3% [15]. These pathogens have severe consequences for human health, including the outbreaks of communicable diseases, diarrhoea epidemics, cholera, skin diseases and other enteric illness. Furthermore, the clinically relevant antibiotic resistance genes were also reported in these pathogens [22]. Their presence in wastewater highlights the negative effects of HWW effluents on ecological balance and in public health.

Globally, several studies have reported on the concentration and fate of antibiotics in water resources and municipal and hospital wastewaters [23,24,25,26]. As a whole, India is reeling under the burden of antimicrobial resistance, including having the highest number of multi-drug resistant tuberculosis in the world [27,28]. Similarly, antimicrobial-resistant bacteria and antibiotic resistance genes have been reported in its natural resources such as water bodies [29]. In particular, resistance toward fluoroquinolones, carbapenem, and colistin, as well as against newer antimicrobials such as carbapenems and faropenem has been reported among Gram-negative and Gram-positive bacteria [4,27]. However, studies of antibiotic concentration and bacterial communities in municipal and hospital wastewater in the State of Tamilnadu, India, are seldom reported. Timely comprehensive surveillance is required if efforts to curtail the environmental spread of ARGs as well as potentially harmful pathogens are to be successful. It is also necessary to determine the role played by the HWW in the spread of antibiotic resistance genes in the environment. Therefore, this study was aimed at extracting and quantifying antibiotic residues in domestic and hospital wastewater effluents, and determining the taxonomic and functional profiles of their bacterial communities using high-throughput sequencing analysis.

## 2. Results

### 2.1. Physicochemical Analysis

The results of the physicochemical characteristics of the wastewater samples are summarised in Table 1. The pH of the water samples fluctuated from slightly acidic (6.6) to neutral (7.2). Other parameters such as conductivity (COND), salinity (SAL), dissolved organic carbon (DOC), dissolved oxygen (DO), and major anions noticeably varied between the wastewater samples. Nutrients such as bromides (Br^−^), nitrites and nitrates were not detected in the hospital wastewater. However, the concentrations of Cl^−^, F^−^ and PO_4_^3−^ were significantly higher (*p* < 0.05) in HWW as compared to DWW samples. The physicochemical parameter readings were compared with the recommended Indian standards for public sewers and United Nations Environment Programme (UNEP) limits. The values for pH, TDS, Nitrate, Nitrite and Sulfate complied with both the Indian standards and UNEP limits. However, Cl^−^, F^−^ and PO_4_^3−^ concentrations in the HWW samples were above the recommended UNEP limits and Indian standards for public sewers, respectively.

### 2.2. Antibiotics Concentration

**Figure 1:**
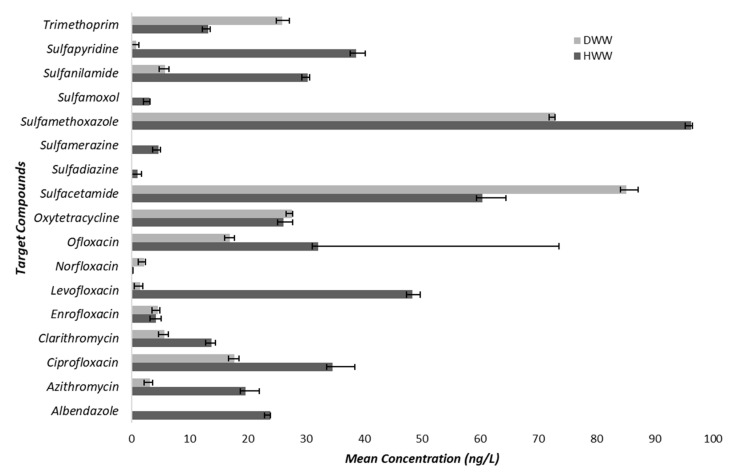
Mean concentration of targeted antibiotic compounds in hospital and domestic wastewater samples. Error bars indicate the 95% confidence intervals.

The chromatograms of the targeted compounds and the summary of mean concentrations are given in Supplementary Table S1 and Figure S1. The linearity range for all the compounds was within 1–1000 µg/L, as determined by the coefficient of determination which equated to r^2^ ≥ 0.99 for all the antibiotics except azithromycin whose value was 0.95. With the exception of norfloxacin and sulfapyridine, all the compounds were detected with mean concentrations higher than 1 ng/L in HWW and DWW, respectively. The other compounds showed varying concentrations as follows; sulfamethoxazole 96.23 ng/L (HWW) v 72.87 ng/L (DWW), levofloxacin 8.27 ng/L (HWW) v 1.47 ng/L (DWW), sulfapyridine 38.66 ng/L (HWW) v 0.88 ng/L (DWW), ciprofloxacin 34.57 ng/L (HWW) v 17.66 ng/L (DWW), ofloxacin 32.13 ng/L (HWW) v 16.97 ng/L (DWW), trimethoprim 13.22 ng/L (HWW) v 25.93 ng/L (DWW), clarithromycin 13.81 ng/L (HWW) v 5.64 ng/L (DWW), and azithromycin 19.66 ng/L (HWW) v 3.21 ng/L (DWW). Compounds such as albendazole, sulfadiazine, sulfamerazine and sulfamoxol were not detected in DWW samples. The Welch *t*-test showed that, except for enrofloxacin, ofloxacin, oxytetracycline and sulfadiazine, the concentrations of all other antibiotic compounds were significantly higher (*p* < 0.05) in HWW than in DWW samples (Figure 1).

### 2.3. Diversity of Bacterial Communities

**Figure 2:**
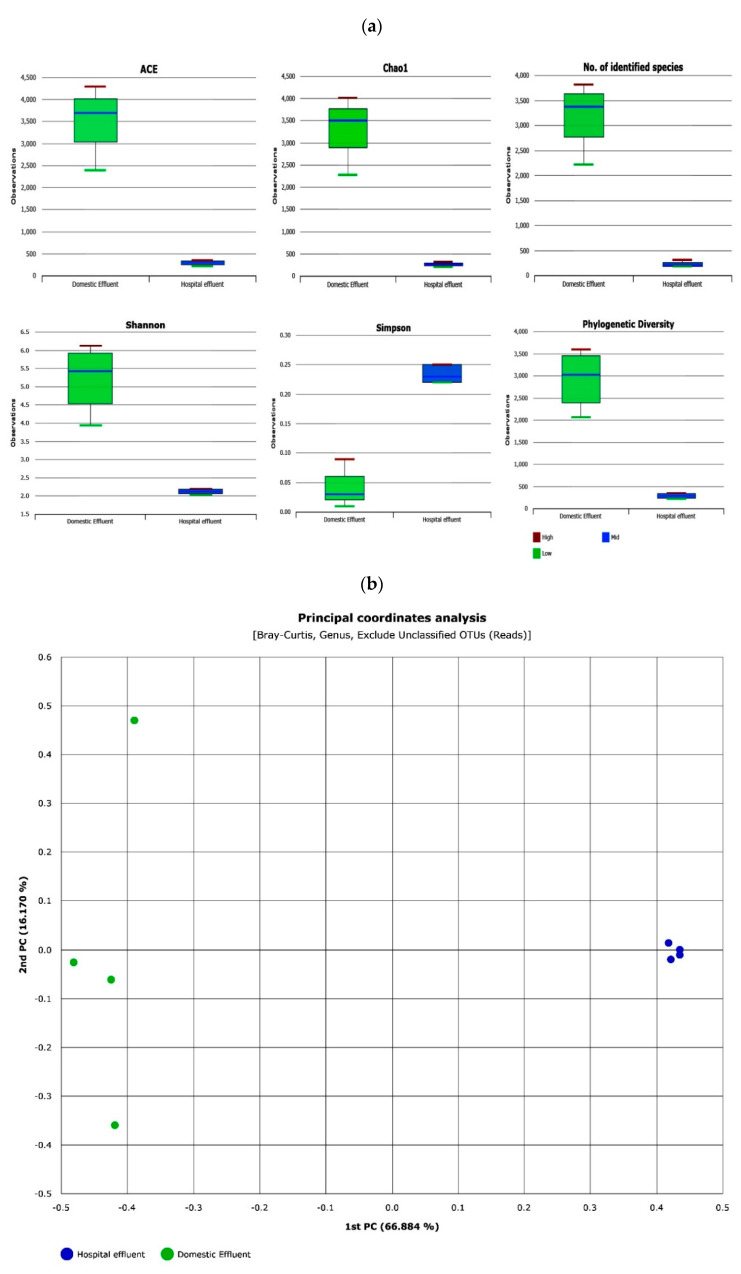
Diversity indices of hospital and domestic wastewater samples (**a**) Box-plots representing the Alpha diversity indices—ACE, Chao1, Shannon and Simpson. Median values (horizontal line) and interquartile ranges have been depicted in the plots. (**b**) Principal coordinate analysis (PCoA) based on Bray–Curtis dissimilarities grouped by effluent samples.

The total valid sequence reads for DWW and HWW were 209,618 and 219,821, respectively, after quality filtering, trimming, and removing all low quality and non -targeted amplicons and chimeric sequences. Further, sequence processing resulted in the assignment of 429,439 high-quality bacterial sequences into 13,706 operational taxonomic units (OTUs). A higher number of OTUs per sequence reads were consistently observed in the DWW system (12,806 OTUs) compared to the HWW system (900 OTUs). In addition, sequences recovered from the two different wastewater system (DWW and HWW) were subjected to alpha-diversity analysis to determine if, indeed, there were more bacterial communities in sample DWW compared to sample HWW. Results indicated that sample DWW harboured significantly higher bacterial diversity (*p* ≤ 0.05) compared to sample HWW. The two diversity indices (Shannon-H and Simpson) are presented in Figure 2a. Good’s coverage of sequence data showed high sequence coverage of 98.98% and 99.39% for DWW and HWW respectively. Similarly, the estimated richness indices ACE and Chao1 showed less OTU diversity and richness in the HWW sample compared to the DWW sample. Beta-diversity-based bacterial composition results were confirmed using principal coordinates analysis (PCoA) Bray–Curtis distance model (Figure 2b), which showed that bacterial communities recovered from the HWW system were clustered together within an ordination, indicating species homogeneity, while samples drawn from the DWW system had significantly diverse bacterial species.

**Figure 3:**
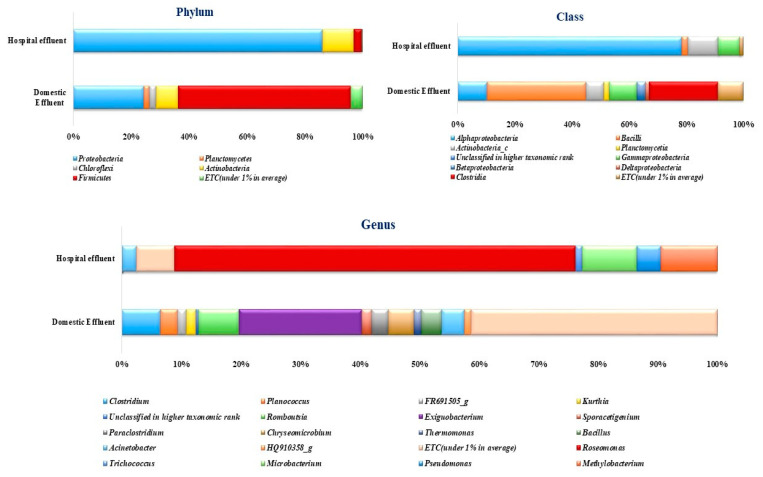
Taxonomic profiling of bacterial communities at the phylum level, class level and genus level in the collected hospital and domestic effluents.

High-throughput sequencing analysis showed that the bacterial communities in the DWW were distributed among five major bacterial phyla. Of these, the phylum *Firmicutes* dominated with 59.61% of the total sequences recovered, followed by *Proteobacteria* (24.48%), *Actinobacteria* (7.66%), *Chloroflexi* (2.29%) and *Planctomycetes* (1.98%). In contrast, few bacterial phyla were identified in the hospital wastewater (HWW) samples, the major phyla being *Proteobacteria* accounting for 86.32% of the recovered sequences, followed by *Actinobacteria* (10.87%) and *Firmicutes* (2.58%). Phylum *Planctomycetes* and *Chloroflexi* were not identified in the HWW samples. Minor phyla, including *Bacteroidetes*, *Fibrobacteres*, *Cyanobacteria*, *Spirochaetes*, *Verrucomicrobia* and *Tenericutes* were detected in DWW and HWW, making up 3.95% and 0.22% of the total recovered sequences, respectively. Detailed metataxonomic profiling of bacterial communities (phylum, class and genus) in the wastewater systems of both the DWW and HWW is presented in Figure 3.

At class level, eight dominant bacterial classes were identified in DWW samples, whereas only four major bacterial classes were identified in HWW samples. Sequences representing the class *Bacilli* were the most abundant in DWW samples, constituting 34.67%, while *Alphaproteobacteria* (78.44%) dominated in HWW samples. Other major bacterial classes identified in DWW included *Clostridia* (24.14%), *Gammaproteobacteria* (9.62%), *Alphaproteobacteria* (10.36%), *Actinobacteria* (6.08%), *Betaproteobacteria* (3.04%), *Planctomycetia* (1.89%) and *Deltaproteobacteria* (1.32%). In HWW samples, the second most abundant bacterial class was *Actinobacteria* (10.58%), followed by *Gammaproteobacteria* (7.45%), and *Bacilli* (2.12%) (Figure 3).

At the genus level, 254 genera were obtained from both samples. Among the assigned genera, 33 were shared by both domestic and hospital wastewater samples. Since the qualities of wastewaters are heterogeneous, a uniformity of genus abundance across wastewaters was unlikely. Therefore, a bacterial genus having a relative gene frequency of >1% was considered. Based on this threshold value, DWW samples were dominated by the genus *Exiguobacterium* (20.65%), *Rombuoutsia* (6.83%), *Clostridium* (6.50%), *Chryseomicrobium* (4.24%), *Acinetobacter* (3.75%), *Bacillus* (3.46%), *Planococcus* (2.86%), *Sporacetigenium* (1.64%), *Kurthia* (1.58%) and *Thermomonas* (1.25%). Meanwhile, HWW samples were dominated by the genus *Roseomonas* (67.41%), followed by the genera *Methylobacterium* (9.47%), *Microbacterium* (9.20%), *Pseudomonas* (3.99%), *Acinetobacter* (2.39%), and *Trichococcus* (1.09%).

**Figure 4:**
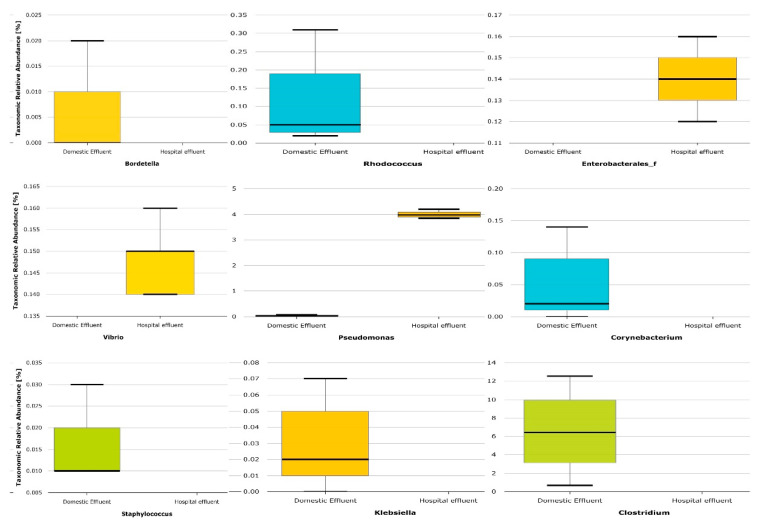
Box plots representing the relative abundance (%) of pathogenic bacterial members in collected wastewater samples. Median values (horizontal line) and interquartile ranges have been depicted in the plots.

Further, the sequences were investigated for *Firmicutes* to *Bacteroidetes* ratio (F/B ratio) and possible pathogens. The F/B ratio was highly significant at *p* = 0.021 between the two samples, showing a ratio of 26:232 to 9:15 for DWW v HWW, respectively. However, the obtained results of F/B ratio in both wastewater was within the optimal ratio of 12–620. Further, the pathogenic bacterial 16S-rRNA-encoding DNA sequences were identified from the major phyla of *Proteobacteria* and *Firmicutes*. The results indicated that the genera *Enterococcus, Pseudomonas* and *Vibrio* were significantly more abundant in HWW compared to DWW samples, while the genera *Clostridium*, *Klebsiella, Corynebacterium, Bordetella, Staphylocccus* and *Rhodococcus* were significantly higher in DWW compared to HWW (Figure 4).

### 2.4. Functional Analysis

**Figure 5:**
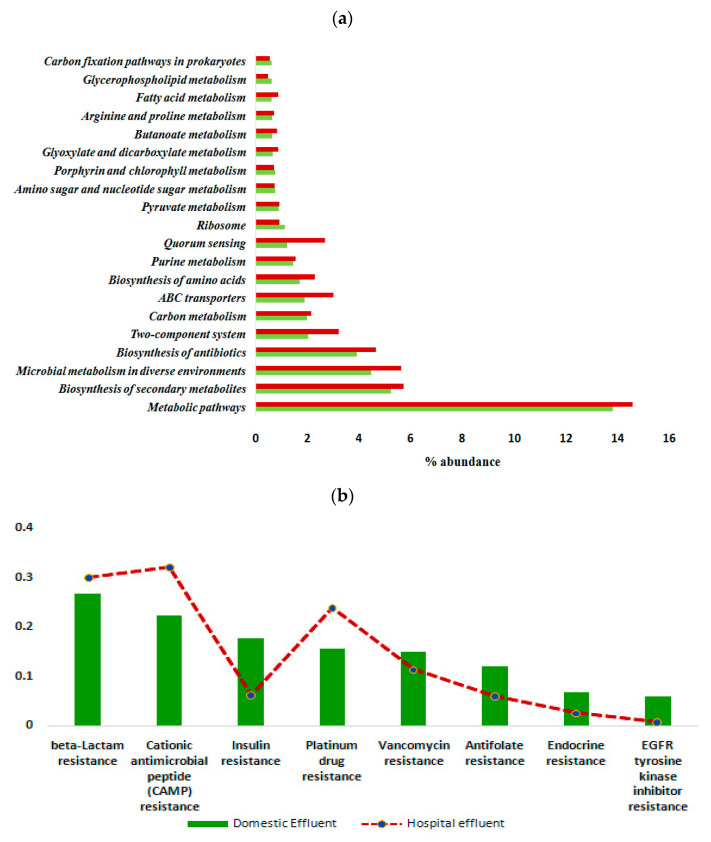
(**a**) Relative abundance (%) of different bacterial pathways detected in the wastewater samples (differences in the overall abundance of metabolic genes in the collected effluent samples were significant; *p =* 0.021). Red bar represented HWW samples and green bar represented DWW samples. (**b**) Various antibiotic resistance pathways identified in domestic and hospital effluent samples.

Predictive functional analysis using PICRUSt2 revealed the presence of 9808 KEGG orthologs (KOs) in domestic wastewater (DWW) samples and 6507 KOs in hospital wastewater (HWW) samples. Identified MetaCyc pathways included 403 and 398 pathways in the DWW and HWW samples, respectively. The nearest-sequenced taxon index (NSTI) values for the predicted enzymes ranged from 0.05–0.35 (data not shown), suggesting a good prediction accuracy. The most abundant and prevailing classification was the metabolic pathway, which included carbohydrate, amino acid, fatty acid and sulphur metabolism. The genes most associated with amino acid metabolic pathways were glycine, serine and threonine metabolism (KO00260), followed by cysteine and methionine metabolism (KO00270) and arginine and proline metabolism (KO00330). Most of the metabolic pathways including fatty acid, lipid, purine and pyruvate metabolism were significantly more pronounced (*p* = 0.021) in HWW samples compared to DWW. Besides the metabolic pathways, genes responsible for quorum sensing, ribosome biogenesis and ABC transporters were also abundantly identified, and had a Benjamin–Hochberg FDR < 0.05 (Figure 5a).

Notably, the resistance pathways included the beta-lactam resistance, cationic antimicrobial peptide (CAMP) resistance, platinum drug resistance, vancomycin resistance, anti-foliate resistance, insulin resistance, endocrine resistance and EGFR tyrosine kinase inhibitor resistance (Figure 5b). Among the resistance genes, antibacterial resistance such as beta-lactam, CAMP, and platinum drug resistance were highly abundant in HWW, while vancomycin resistance dominated in DWW samples. Expression of ARGs encoding efflux pumps was significantly lower (*p* < 0.05) in DWW compared to HWW. Notable signature modules were beta-lactam resistance (M00851), vancomycin resistance, D-Ala-D-Lac type (M00651), as well as multidrug resistance, efflux pumps MexAB-OprM (M00718), MexCD-OprJ (M00639), BpeEF-OprC (M00698), NorB (M00702) and QacA (M00714). These efflux pumps were associated with *Enterobacteriaceae*, *Enterococcaceae, Pseudomonadaceae, Klebsiella, Acinetobacter, Staphylococcaceae* and *Bacillaceae*, respectively.

## 3. Discussion

Environmental disposal of untreated wastewater creates severe challenges to public and environmental health. However, as per the data in Table 1, the pH of the wastewater samples, which ranged from slightly acidic (6.6) to neutral (7.2) is similar the pH range reported in previous studies [30,31,32]. Importantly, the recorded pH values did not deviate from the World Health Organization (WHO) pH tolerance limit of between 6.00–9.00 for wastewater to be discharged into streams and rivers. While ammonia was detected in both DWW (0.75) and HWW (0.13), nutrients such as nitrites and nitrates were not detected in HWW. Literature evidence suggests that the presence of antibiotic compounds in the environment is detrimental, as they may kill off nitrifying and denitrifying bacteria, impacting the process of nitrogen fixation in the terrestrial environment [33] while at the same time exerting a selective pressure that favors the proliferation of antibiotic-resistant bacterial communities [34,35]. The concentration of chloride and fluoride ions in HWW exceeded the EPA permissible limits as well as the Indian standards for wastewater discharge. From an environmental health perspective, these fluorides are common contaminant in different industrial wastewaters [2], and are reported to inhibit different microbial activities including methane production, nitrification, glucose fermentation and few degradation pathways [36]. The levels of other chemical parameters shown in Table 1 were within the permissible limits and were judged to be of no major consequence in shaping the taxonomic and functional profiles of bacterial communities in the studied wastewaters.

Despite widely reported health issues being linked to antibiotics in wastewater, to the authors’ knowledge, in India very few studies attempted the quantitative estimation of antibiotics in domestic and hospital wastewater [37,38], and none of the studies report any findings from Tamil Nadu. Data in Table S1 illustrates the mean concentration of different antibiotics quantified in domestic and hospital wastewater. Except for norfloxacin (0.28 ng/L), all other detected compounds showed a mean concentration higher than 1 ng/L in hospital wastewater, which is not consistent with the findings of Diwan et al. [37], who reported norfloxacin concentrations of between 5.7 and 22.8 ng/L in different hospital wastewater samples. The observed high concentrations of ciprofloxacin, ofloxacin, oxytetracycline, sulfamethoxazole, sulfanilamide and trimethoprim in both wastewater samples reflects a society with a high dependency on medication, even if used at home or in hospitals. A similar observation was reported in Portugal and Italy, where high concentrations of pharmaceuticals were found in different hospital and WWTPs effluents [39,40]. Among the antibiotics reported in these studies, the most prevalent were ofloxacin, ciprofloxacin, sulfamethoxazole, and clarithromycin. This is not surprising because these antibiotics are first-line antibiotics for different infections. For instance, the combination of trimethoprim/sulfamethoxazole (TMP/SMX) is used as a first line antibiotic to treat a range of bacterial infections including urinary tract infections (UTIs), methicillin-resistant *Staphylococcus aureus* (MRSA) skin infections, travellers’ diarrhoea, respiratory tract infections, and cholera [41]. Similarly, either in combination or in singular form, the antibiotics ciprofloxacin and ofloxacin are/is used to treat bacterial infections such as community-acquired pneumonia, tuberculosis, bronchitis, staphylococcus infections, sexually transmitted diseases (STDs), UTIs, and prostate infections caused by *E. coli* [42,43]. Similar to other studies, this study also confirms the detection of antibiotic residues in relatively higher concentrations in HWW than DWW [44,45]. In addition, the different profiles of antibiotics used in hospitals compared to home settings (Figure 1) could have influenced the different bacterial profiles found in HWW compared to DWW (Figure 4). In previous studies, this has been attributed to the more frequent use of glycopeptides and carbapenems in hospitals than in home settings [46,47]. Besides the increased antibiotic resistance among bacterial populations found in HWW as compared to DWW, bacterial species diversity was lower in HWW compared to DWW (Figure 4), possibly due to antibiotic action on the susceptible populations. Such higher concentrations of pharmaceuticals in the environment provide ideal conditions for development of antibiotic resistance within the resident microbes, and spread of resistant pathogens leading to complex cross-selection patterns that constitute challenges for public health [48]. Several authors have reported the occurrence of antibiotic-resistant bacteria as well as antibiotic resistance genes in underground water sources [45,49], with the risk of subsequent transfer of antibiotic resistance from environmental strains to normal bacterial flora in the human gut upon drinking of that water [50]. This is a critical finding given the proportion of people belonging to the state of Tamil Nadu depending on underground water for daily consumption and given the possible recharge of those underground water bodies by water percolating from the polluted Koovam River.

The results of diversity indices showed that hospital wastewater displayed less richness (Chao 1) and diversity (Simpson) compared to domestic water, similar to observed trends in the samples collected at the medical centre located in Daegu, South Korea [18]. This suggests that higher concentrations of antibiotics affect bacterial population dynamics as much as it promotes dissemination of antibiotic resistance [51]. Despite the observed bacterial phyla richness among the samples analysed in this study, as in other studies also, it is either the *Proteobacteria* or the *Firmicutes* whose predominance has been observed across a range of wastewater samples [2,32,52,53]. The dominance of these two phyla could be correlated to their capacity to survive in extreme environmental conditions and high contaminant levels [1], and their possible resistance against the detected antibiotics. Notably, sequences representing the phylum *Bacteroidetes* were less than 1% of total recovered sequences in both samples, which is contradictory to other reports [54,55] where its sequences accounted for a higher percentage of recovered sequences. Within the bacterial classes, *Alphaproteobacteria* was the most abundant class, with HWW having the highest abundance of 78.44%, and DWW having the lowest abundance (10.34%), suggesting the observed differences in the structure of bacterial communities between wastewater samples can probably be attributed to various factors such as nutrient composition, anthropogenic disturbance and other physicochemical conditions [56].

Among the identified genera, domestic wastewater was dominated by the genus *Exiguobacterium*, which has previously been identified as a dominant member of both upstream and downstream samples of a river influenced by a Wastewater Treatment Plant [19]. Despite the bacterium being widely distributed in diverse environments [57], it has a potential to cause community-acquired pneumonia (CAP) and bacteraemia in a diabetes patients [58]. Likewise, the genus *Roseomonas* was dominant in hospital wastewater, as also observed by Cecilia et al. [32], who profiled the bacterial diversity in different wastewater treatment plants. A previous study demonstrated that *Roseomonas* species are now increasingly being referred to as evolving opportunistic pathogens for their connection to human infectious diseases [59]. Interestingly, the abundance of *Acinetobacter,* a frequent causative agent of nosocomial pneumonia was higher in DWW than in HWW samples, when one may have expected it to be vice-versa. Reports have even indicated that the species *Acinetobacter baumannii* can cause several infections including skin and wound infection, infective endocarditis, bacteraemia, UTIs, and meningitis [60,61]. It has also been found to be resistant to many of the antibiotics and currently poses one of the greatest hazards to public health [62]. Other notable genera identified in HWW include *Methylobacterium, Microbacterium, Pseudomonas* and *Trichococcus* (Figure 3). According to several reports, these are commonly found bacterial isolates in hospital effluents, and have been shown to exhibit multiple antibiotic resistance [1,16,63,64]. Infections caused by these multi drug-resistant organisms are often fatal in people with other underlying conditions such as diabetes, hypertension, obesity, cardiovascular disease, asthma, kidney disease or chronic obstructive pulmonary disorder. Therefore, this study also focussed on *Firmicutes/Bacteroidetes* ratio (Supplementary Figure S2), a specific microbial signature, more particularly for those associated with obesity, type 2 diabetes, and cardiovascular diseases [65]. The results were within the optimal *Firmicutes/Bacteroidetes* ratio suggesting either that a significant percentage of the Tamil Nadu population do not have chronic health conditions or that there are more younger people than elderly, since the *Firmicutes/Bacteroidetes* ratio tends to alter with age [66,67].

The pathogenic bacterial sequences identified in this study included some of the most common pathogenic bacteria, which could be used for microbial source tracking, including *Enterococcus, Pseudomonas*, *Vibrio,* *Klebsiella, Corynebacterium, Bordetella and Staphylocccus* in both the HWW and DWW samples. The members of *Enterobacteriaceae* family including *Enterococcus* and *Klebsiella* produce an extended spectrum of beta-lactamases (ESBLs) that are capable of hydrolysing a wide range of antibiotics [68], which in this study was confirmed by the detection beta-lactam resistance signature modules and vancomycin resistance, D-Ala-D-Lac in HWW samples, respectively (Figure 5b). Notably, bacteria of the genus *Enterococcus* are the major causes of nosocomial infections, and the prevalence of vancomycin resistance has increased in recent decades [69], probably due to high antimicrobial pressure in the environment. As expected, bacteria of the genus *Pseudomonas* were high in HWW. *Pseudomonas* is reported as a frequent pathogen in hospital water networks and water points compared with urban/domestic wastewater [70]. This study also confirms the presence of MexAB-OprM efflux pump of *Pseudomonas*, which is one of the largest multi-drug resistant efflux pumps with high-level expression. This arrangement would allow the intrinsic resistance of *Pseudomonas* to different classes of antibiotics and its ability to acquire resistance to almost all effective antibiotics, which may complicate the treatment of infections. Sequences belonging to another pathogenic genus, *Vibrio*, were also high in HWW. Despite its pathogenic nature, recent trends shows that most of the clinical isolates of *Vibrio* are resistant against almost all routinely used antibiotics [71]. Similarly, the pathogen *Klebsiella* whose sequences were dominant in DWW samples is also reported as multidrug-resistant, and known to cause significant morbidity and mortality worldwide [72]. Genera such as *Corynebacterium* and *Bordetella* are conventional pathogens that can be found in symptomless carriers; however, these carriers can give rise to an outbreak of disease in a healthy community. Sequences of another pathogen, this time of the genus *Staphylococcus*, were high in DWW and functional analysis showed that *Staphylococcus* carry the multidrug resistance, efflux pump QacA (Figure 5b). There is evidence that the plasmid-encoded multidrug resistance gene QacA from *Staphylococcus aureus* stimulates high efficiency of drug extrusion and mediates resistance to a variety of antimicrobial agents [73,74]. Although this study has demonstrated the significant correlation between predicted multi-drug resistance genes and bacterial pathogens, further validations are warranted using culture-based approaches or functional metagenomics.

## 4. Materials and Methods

### 4.1. Study Area and Sampling

Wastewater samples comprising of domestic and hospital wastewater were collected from two different locations in Chennai City and Tamil Nadu, India. Both domestic wastewater (DWW) and hospital wastewater (HWW) effluents are discharged into the river Koovam that flows through three corporation zones for a total length of 16 kilometres. The river is therefore highly polluted until its mouth in the Bay of Bengal due to continuous discharge of untreated wastewater effluents. Compounding the pollution crisis is about 3500 illegal hutments that have been built along the banks of the river, which also discharge large volumes of untreated DWW. Critically, people living in the vicinity of the Koovam River rely on groundwater sources for daily use [75]. Four DWW discharge points along the densely populated Koovam River banks were chosen for collection of DWW samples while HWW samples were collected from four different discharge points of the Government General Hospital, where about 12,000 to 15,000 outpatients receive treatment daily. Wastewater samples were collected into two litre sterile sampling bottles containing 1.67 mL of 10% sodium thiosulphate as standard practice [76]. Wastewater samples were immediately transported to the laboratory in cooler boxes containing ice and analysed within 12 h of collection.

### 4.2. Physico-Chemical Analysis of Wastewater Samples

Some physico-chemical parameters including pH, dissolved oxygen (DO), conductivity, salinity, ammonia nitrogen (NH_3_-N) and total dissolved solids (TDS) were measured in situ during sampling using a YSI professional plus (Xylem Inc., Yellow Springs, OH, USA) instrument. The methods earlier reported by Haile et al. [31] were used to determine the anions and Dissolved Organic Content (DOC). Briefly, for anionic determination, the samples were pre-filtered using 0.45 µm syringe filters with GHP membranes (PALL life sciences, Ann Arbor, MI, USA) and injected into a Metrohm ion chromatograph 861 (Herisau, Switzerland) equipped with a conductivity detector. A multi-anionic standard solution (PerkinElmer, Spokane, WA, USA) containing the target anions with stock concentrations of 100 mg L^−1^ was used to prepare calibration curves. The separation was carried out on a Metrosep A supp 5 (250 × 4 mm^2^) anion exchange column and an IC.Net 2.3 (Metrohm) software was used for data acquisition and data analysis. For DOC analysis, 3 mL of filtered samples were injected into a TOC analyser equipped with an autosampler and high-pressure Non-Dispersive Infrared (NDIR) detector (Torch TOC/TN, Teledyne Tekmar, Mason, OH, USA). A Six-point calibration curve was constructed using standard solutions ranging from 0 to 20 mg L^−1^ of potassium hydrogen phthalate (KHP) to determine the exact concentration.

### 4.3. Extraction and Quantification of Antibiotics

Seventeen antibiotic compounds were targeted for detection in this study: albendazole, azithromycin, ciprofloxacin, clarithromycin, enrofloxacin, levofloxacin, norfloxacin, ofloxacin, oxytetracycline, sulfacetamide, sulfadiazine, sulfamerazine, sulfamethoxazole, sulfamoxol, sulfanilamide, sulfapyridine and trimethoprim. The extraction and quantification of antibiotics was carried out following the method proposed by Mhuka et al. [77]. Briefly, the collected water samples were extracted in Dionex AutoTrace™ automated SPE (Dionex™ AutoTrace™, Thermo Scientific, Braunschweig, Germany) Unit using Waters Oasis^®^ HLB solid phase extraction (SPE) cartridges (Waters Corporation, Milford, MA, USA). Prior to the extraction process, the cartridges were pre-conditioned with methanol and after extraction, the cartridges were washed with 5% methanol in water, and subsequently dried under vacuum for 20 min. The dried extracts were completely evaporated using a stream of nitrogen and then reconstituted in 1 mL of methanol for analysis. A Thermo Scientific™ Q Exactive™ Plus Orbitrap™ Mass Spectrometer coupled to a Thermo Scientific™ Dionex UltiMate™ 3000 UHPLC system (Thermo Fisher Scientific, Waltham, MA, USA) was used for quantification. A Waters^®^ XBridge™ C18 (2.1 × 100 mm^2^, 3.5 mm particle size) HPLC column was used with 0.1% (*v*/*v*) formic acid in water as mobile phase A and 0.1% (*v*/*v*) formic acid in acetonitrile as mobile phase B. The linear gradient elution was adopted by adjusting the column temperature at 30 °C. Five microliters (5 µL) of sample was injected with a flow rate of 0.3 mL/min and a total run time of 21.5 min. Finally, data processing was performed using TraceFinder™ EFS Software Version 3.2 (Thermo Fisher Scientific Inc., Waltham, MA, USA).

### 4.4. DNA Extraction, Library Preparation and Illumina Miseq High Throughput Sequencing

Total DNA was extracted from 5 mL of each collected sample using a Faecal/Soil Total DNA™ extraction kit (Zymo Research Corporation, Irvine, CA, USA) according to the manufacturer’s instructions. The resultant DNA concentration and quality were checked at 260 nm wavelength and absorbance ratios of 260/280 nm on a NanoDrop Spectrophotometer (NanoDrop Technology, Wilmington, DE, USA) following which the DNA was preserved at −20 °C until further processing. The extracted DNA was first amplified using the universal bacterial 16S rRNA primers (27F and 1492R) to cover the whole variable region under the following PCR conditions: initial denaturation at 95 °C for 5 min, followed by 32 cycles of melting at 95 °C for 1 min, annealing at 55 °C for 1 min, and elongation at 72 °C for 1 min. This was then superseded by a final elongation step at 72 °C for 10 min. Subsequently, a second PCR run was carried out using the 27F and 518R primer sets, with overhanging adapter sequences that are compatible with Illumina index as described by Ramganesh et al. [2]. Cleaning of the resultant PCR products, index library preparation, pooling and sequencing on Illumina Miseq 250^®^ to generate paired 300-bp high-quality reads of the V1–V3 region were performed according to standard protocol (Illumina Inc., San Diego, CA, USA).

### 4.5. Sequence Data and Statistical Analysis

Following sequencing, the raw sequence datasets were initially scrutinised for PCR artifacts and low-quality reads using an *ngsShoRT* (next-generation sequencing Short Reads) trimmer as described by Chen et al. [78]. Primers were trimmed using Chunlab in-house program (Chunlab, Inc., Seoul, Korea) at a similarity cut-off of 0.8. The sequences were de-noised using the DUDE-Seq to correct sequencing errors. The quality-controlled sequences were then subjected to UCHIME to identify and remove chimera reads and then the non-chimeric sequences were subjected to classification using 16S database in the EzBioCloud to determine taxonomic assignment. Sequences that matched the reference sequence by more than 97% similarity in EzBioCloud were considered identified at the species level [79]. Nonparametric diversity indices including Shannon–Weaver index and the Chao1 richness estimator were calculated at the genetic distance of 0.03 to measure the diversity of bacterial species among the data sets. Sample coverage values were calculated by using Good’s formula [80]. The percentage of relative abundance of individual taxa within each community was estimated by comparing the number of sequences assigned to a specific taxon against the total number of sequences obtained for that sample. Principal Coordinate analysis (PCoA) was computed based on Bray–Curtis (BC) dissimilarity after removing the unclassified sequences. The Phylogenetic Investigation of Communities by Reconstruction of Unobserved States (PICRUSt2) software package was used to predict and understand the potential functional capabilities of the bacterial communities and presence of antibiotic resistance genes, as described elsewhere [81]. Normalisation, prediction, and detection of gene pathways was carried out following the method described by Sibanda and Ramganesh [82]. Welch’s *t*-test was used to compare the significance difference between the concentrations of antibiotics in collected wastewater samples, and boxplots were created using the R statistical software [83]. Finally, the obtained sequence and metadata used in this study were deposited into the NCBI’s sequence read archive (SRA) database under BioProject PRJNA746090.

## 5. Conclusions

Our results pinpoint both domestic and hospital wastewaters as sources of antibiotic residues and potentially pathogenic bacterial species in the aquatic environment. The presence of antibiotics such as ofloxacin, ciprofloxacin, sulfamethoxazole and clarithromycin in environmental milieu, even in trace concentrations, provides ideal conditions for evolution of antibiotic resistance within the resident microbes. This, combined with the presence of clinical pathogens such as *Enterococcus*, *Pseudomonas*, and *Vibrio* (in hospital wastewater) and *Klebsiella*, *Corynebacterium*, *Bordetella* and *Staphylocccus* (in domestic wastewater) provides for the emergence of bacterial super-bugs, which might present humanity with larger challenges than presently anticipated. This result is even more marked given that the presence of these pathogens positively correlated with the presence of antibiotic resistance genes and cassettes. These results are very significant in the context of Tamil Nadu given its high population density, and the people’s dependence on groundwater for daily use, which might increase exposure and compromise their health. To alleviate the inevitable, this study suggests an urgent need to establish pathogen surveillance, and appropriate guidelines to mitigate the risk of anti-microbial resistance through the effluent discharge in order to safeguard public and environmental health.

## Figures and Tables

**Table 1:** Physico-chemical characteristics of the wastewaters studied (mean ± SD).

	Domestic Effluent (DWW)	Hospital Effluent (HWW)	UNEP	ENVIS
pH	6.6 ± 0.07	7.2 ± 0.28	6.6–8.5	6.5–8.5
DO (mg L^−1^)	0.534 ± 0.50	0.94 ± 0.42	-	-
Conductivity (μS/cm)	902 ± 2.21	628 ± 6.36	380	-
Salinity (SAL)	0.45 ± 0.07	0.3 ± 0.00	-	-
NH_3_-N	0.745 ± 0.008	0.13 ± 0.04	0.21	-
DOC (mg L^−1^)	50.39 ± 0.62	67.35 ± 0.21	-	-
TDS (mg L^−1^)	587.5 ± 0.71	431.5 ± 18.68	-	1500
Cl (mg L^−1^)	19.64 ± 3.75	32.95 ± 2.82	20	-
Br^−^ (mg L^−1^)	0.45 ± 0.095	ND	-	-
F^−^ (mg L^−1^)	0.42 ± 0.081	4.58 ± 0.52	-	1.5
Nitrite (mg L^−1^)	0.425 ± 0.130	ND	-	20
Nitrate (mg L^−1^)	0.31 ± 0.05	ND	0.16	50
Sulfate (mg L^−1^)	29.68 ± 1.48	19.58 ± 0.36	500	400
Phosphate (mg L^−1^)	5.32 ± 0.036	19.69 ± 0.57	4.5	10

UNEP—United Nations Environment Programme; ENVIS—Indian standard for public sewers; ND—not-detected.

## Data Availability

The obtained sequence and metadata used in this study were deposited into the NCBI’s sequence read archive (SRA) database under BioProject PRJNA746090.

## Supplementary Materials

The following are available online at https://www.mdpi.com/article/10.3390/antibiotics10091059/s1, Figure S1: Observed chromatograms for hospital and domestic wastewater samples, Figure S2: Firmicutes to Bacteroidetes (F/B) ratio of domestic and hospital effluent samples. Table S1: Summary of occurrence and concentration of antibiotics in collected wastewater samples.
