# Supplementary material for: Taxonomic and Functional Distribution of Bacterial Communities in Domestic and Hospital Wastewater System: Implications for Public and Environmental Health
Source: Antibiotics (Basel). 2021 Sep 1;10(9):1059. doi: 10.3390/antibiotics10091059 (PMC8470611; doi:10.3390/antibiotics10091059)

antibiotics

Supplementary Information

Figure S1: Observed chromatograms for hospital and domestic wastewater samples

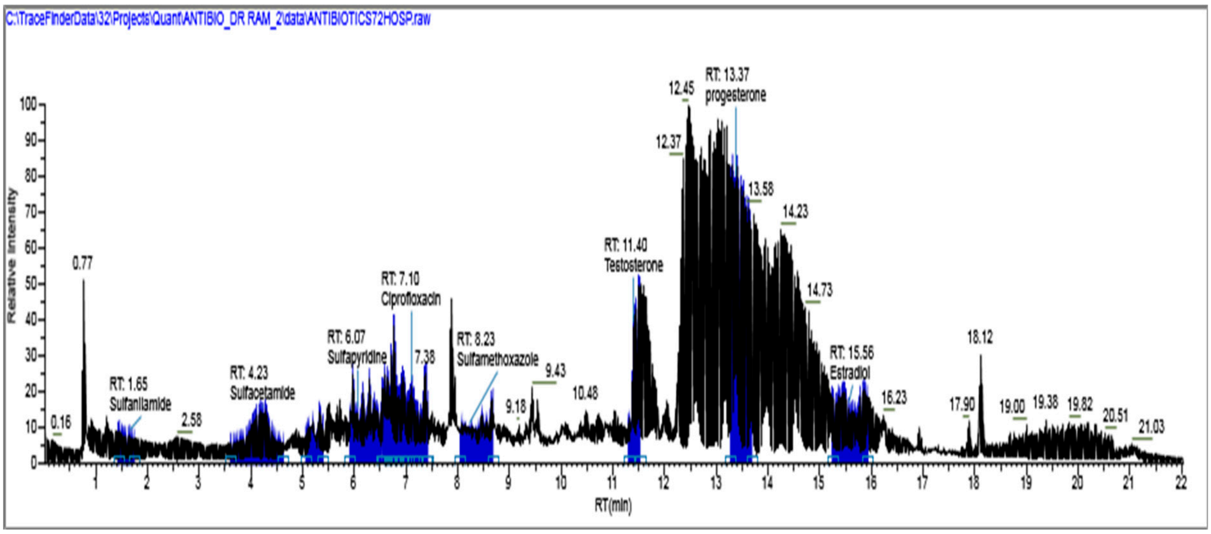

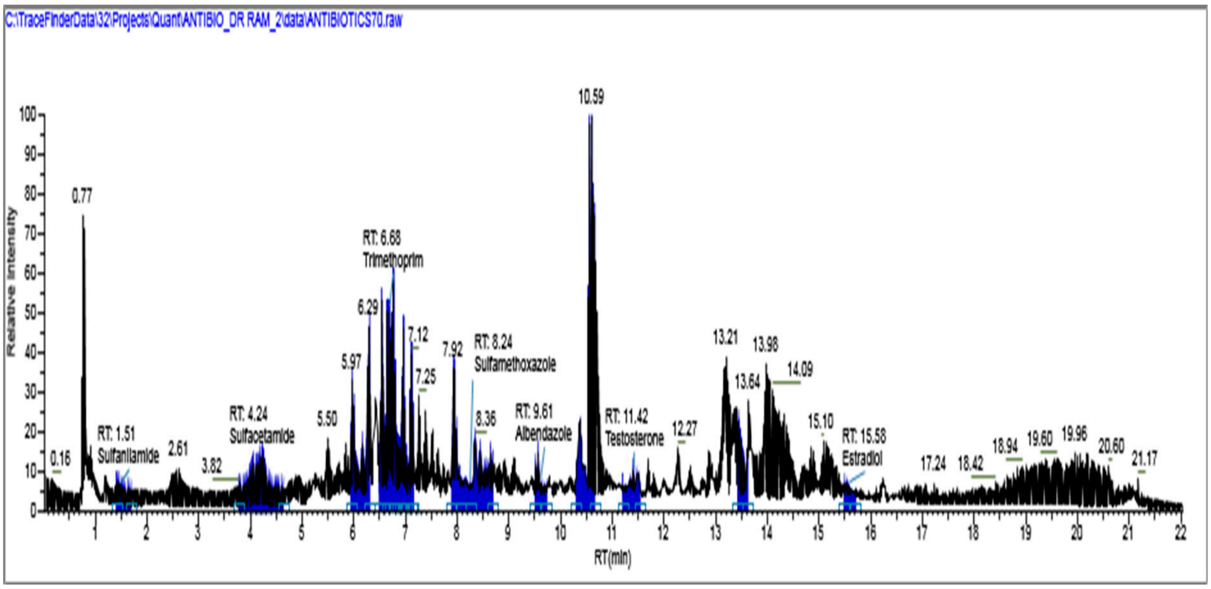

Table S1: Summary of occurrence and concentration of antibiotics in collected wastewater samples

S/N	Antibiotics	Mean concentration (ng/L) HWW	Mean concentration (ng/L) DWW	t-stat	p-value
1	Albendazole	23.85	0	542.09	0.000*
2	Azithromycin	19.66	3.21	10.06	0.009*
3	Ciprofloxacin	34.57	17.66	6.013	0.027*
4	Clarithromycin	13.81	5.64	12.88	0.002*
5	Enrofloxacin	4.23	4.58	-0.54	0.643
6	Levofloxacin	48.27	1.47	46.09	0.000*
7	Norfloxacin	0.28	2.18	-10.29	0.009*
8	Ofloxacin	32.13	16.97	0.52	0.655
9	Oxytetracycline	26.13	27.63	-1.34	0.309
10	Sulfacetamide	60.34	85.10	-7.61	0.017*
11	Sulfadiazine	1.05	0	2.05	0.176
12	Sulfamerazine	4.62	0	19.29	0.003*
13	Sulfamethoxazole	96.23	72.87	114.91	0.000*
14	Sulfamoxol	3.06	0	32.09	0.001*
15	Sulfanilamide	30.28	5.74	44.97	0.000*
16	Sulfapyridine	38.66	0.88	33.67	0.001*
17	Trimethoprim	13.22	25.93	-14.66	0.005*

* Welch T-test for abundance of antibiotics was used. $P<0.5$ shows significance

Figure S2: Firmicutes to Bacteroidetes (F/B) ratio of domestic and hospital effluent samples.

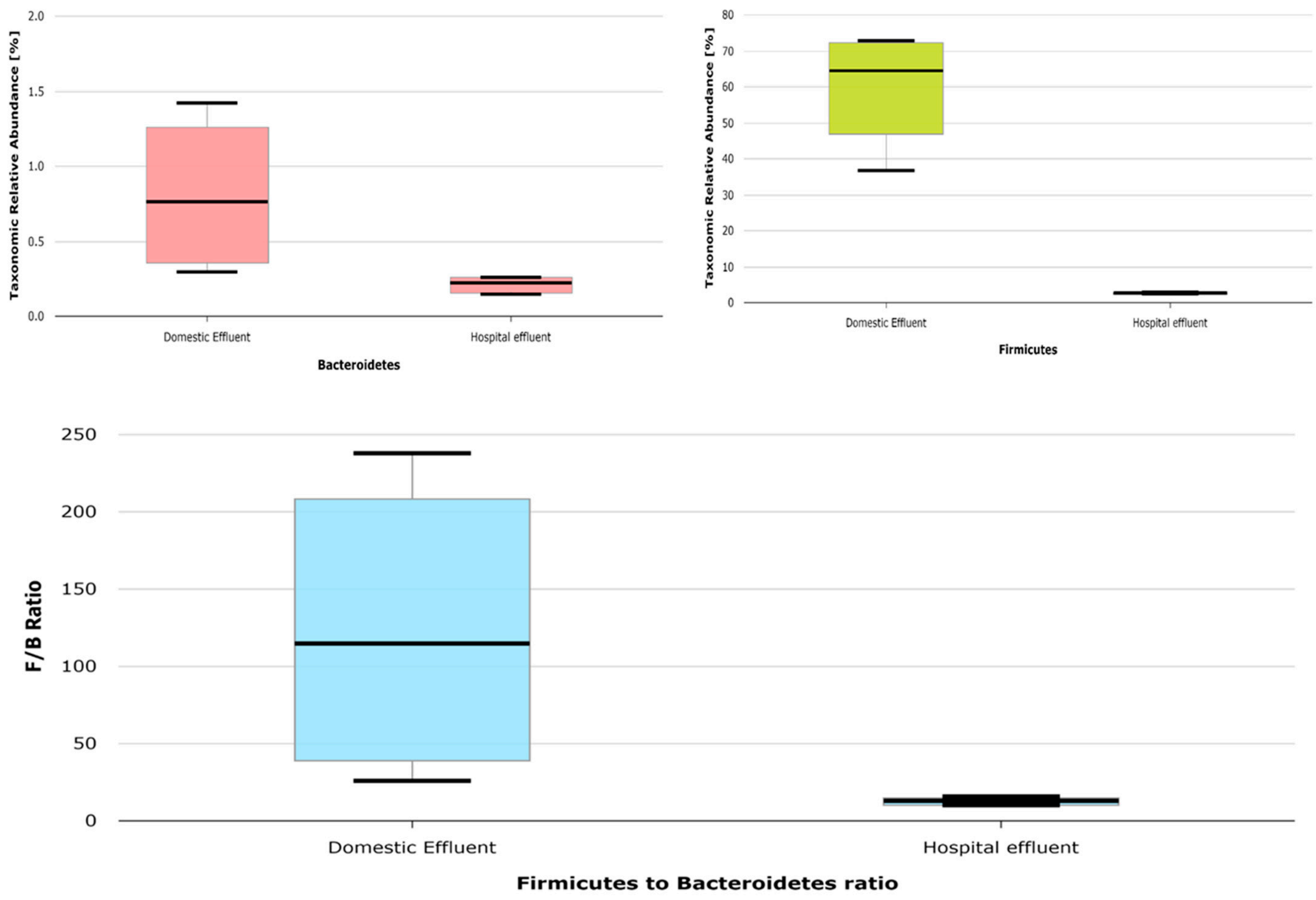

Supplement: Supplementary file 1 [file antibiotics-10-01059-s001.zip › antibiotics-1319333-supplementary.pdf]
